# Comparison of human T cell leukemia virus-1/2 (HTLV-1/2) screening assays in South America: Implications in the loss of blood units

**DOI:** 10.1186/1742-4690-11-S1-O16

**Published:** 2014-01-07

**Authors:** Gabriela Pataccini, Camila Cánepa, Jimena Salido, Cecilia M Delfino, Jorgelina Blejer, Roberto Fernández, Eulalia Rodríguez, Adriana Alter, Williams Pedrozo, Richard Malan, Elida Iriarte, Gustavo Duarte, Vanessa Schneider, Sergio Bontti, Cristina Salomón, Marcelo Borda, Mirna M Biglione, Carolina A Berini

**Affiliations:** 1 Instituto de Investigaciones Biomédicas en Retrovirus y SIDA (INBIRS). Facultad de Medicina, Universidad de Buenos Aires, Buenos Aires, Argentina; 2 Cátedra de Inmunología, Facultad de Bioquímica, Química, Farmacia y Biotecnología. Universidad Nacional de Tucumán, San Miguel de Tucumán, Argentina; 3 Área Serología, Fundación Hemocentro Buenos Aires, Buenos Aires, Argentina; 4 Servicio de Hemoterapia, Sanatorio Municipal “Dr. Julio Méndez”, Buenos Aires, Argentina; 5 Banco de Sangre Central de la Provincia de Misiones, Posadas, Misiones, Argentina; 6 Sanatorio Adventista del Plata, Libertador San Martín, Entre Ríos, Argentina; 7 Servicio de Hemoterapia, Hospital Alemán, Buenos Aires, Argentina; 8 Laboratorio de Referencia de Enfermedades Transmisibles “Centro E. Coni”, Mendoza, Argentina; 9 Facultad de Ciencias Exactas y Naturales, Universidad Nacional del Nordeste, Corrientes, Argentina

## 

Although the detection of HTLV antibodies became mandatory in Argentinean blood banks since 2005, there is no recommendation on how to perform the screening. As a consequence, there is a variable number of HTLV false positives depending on the kit used. The aim of the present study was to evaluate the performance of six commercial screening tests available currently for the initial diagnosis of HTLV-1/2 infection in South America. The positive panel included 14 HTLV-1 and 13 HTLV-2 samples confirmed by WB (HTLV-1/2 MP Diagnostics). The negative panel included 233 samples analysed with Architect (n=47), Diapro (n=46), MP (n=45), Murex (n=47) and Serodia (n=48), collected in 5 different institutions in a period of one up to 10 consecutive days. All of them were confirmed by n-PCR. The sensitivity for all HTLV diagnostic kits was 100.0%. The most specific test was Serodia (PA) (99,1%; 2/233), followed by the ELISAs: Celquest (98.3%, 4/233), Murex (97.8%; 5/233), MP and Architect (97.4%; 6/233), and Diapro (93,1%; 16/233). According to this data, 2 to 16 blood units out of 233 should have been discarded due to false positive results in a period of 10 days. This data must be considered when choosing the assays, not only to obtain an optimal efficiency on HTLV-1/2 diagnosis, to increase the number of potential blood units and to decrease the circulation of anxious individuals without a final result, but also to lower the overall cost-benefits of the diagnosis in the health care system.

